# Distribution of *Rotavirus alphagastroenteritidis* Strains in Blantyre, Malawi, During and After the COVID-19 Pandemic

**DOI:** 10.3390/pathogens14111169

**Published:** 2025-11-16

**Authors:** End Chinyama, Chimwemwe Mhango, Rothwell Taia, Landilani Gauti, Jonathan Mandolo, Flywell Kawonga, Ernest Matambo, Prisca Matambo, Innocent Chibwe, Richard Wachepa, Nigel A. Cunliffe, Chisomo L. Msefula, Khuzwayo C. Jere

**Affiliations:** 1 Malawi–Liverpool–Wellcome Programme, Blantyre 312225, Malawi; cmhango@mlw.mw (C.M.); lgauti@mlw.mw (L.G.); jmandolo@mlw.mw (J.M.); fkawonga@mlw.mw (F.K.); ematambo@mlw.mw (E.M.); pmatambo@mlw.mw (P.M.); inno.chibwe@gmial.com (I.C.); rwachepa@mlw.mw (R.W.); nigelc@liverpool.ac.uk (N.A.C.); 2 Department of Pathology, School of Medicine and Oral Health, Kamuzu University of Health Sciences, Blantyre 312225, Malawi; cmsefula@kuhes.ac.mw; 3 Biomedical Sciences Department, School of Life Sciences and Allied Health Professions, Kamuzu University of Health Sciences, Blantyre 312225, Malawi; 4 Department of Clinical Infection, Microbiology and Immunology, Institute of Infection, Veterinary and Ecological Sciences, University of Liverpool, Liverpool L69 7ZX, UK; taia.rothwell5@nhs.net; 5 Center for Clinical and Biological Sciences Research (CCBSR), Academy of Medical Sciences, Malawi University of Science and Technology, Thyolo 310105, Malawi; 6 Wits African Leadership in Vaccinology Expertise (Wits-Alive), School of Pathology, Faculty of Health Science, University of the Witwatersrand, Johannesburg 2050, South Africa; 7 Department of Clinical Sciences, University of Liverpool School of Tropical Medicine, Liverpool L35QA, UK; 8 Department of Medical Laboratory Sciences, School of Life Sciences and Allied Health Professions, Kamuzu University of Health Sciences, Blantyre 312225, Malawi; 9 Department of Pharmacy, School of Life Sciences and Health Professions, Kamuzu University of Health Sciences, Blantyre 312225, Malawi

**Keywords:** *R. alphagastroenteritidis* infection, *R. alphagastroenteritidis* vaccines, genotypes, gastroenteritis, Malawi, COVID-19 pandemic

## Abstract

*Rotavirus alphagastroenteritidis* remains the leading cause of severe gastroenteritis in children under five years, despite widespread vaccine use. The COVID-19 pandemic disrupted healthcare and vaccination delivery, while non-pharmacological interventions may have influenced *R. alphagastroenteritidis* transmission. We conducted hospital-based surveillance of *R. alphagastroenteritidis* gastroenteritis at Queen Elizabeth Central Hospital (QECH) in Blantyre, Malawi, from October 2019 to October 2024. Children under five presenting with acute gastroenteritis were enrolled; 99.1% of vaccine-eligible participants had received at least one *R. alphagastroenteritidis* vaccine dose. Stool samples were tested for *R. alphagastroenteritidis* by enzyme immunoassay (EIA) and genotyped using RT-PCR. Among 1135 enrolled children, 29.1% (330/1135) were *R. alphagastroenteritidis*-positive. Cases occurred year-round except for December 2020–January 2021, when no *R. alphagastroenteritidis* infections were detected, and February–March 2023, when no samples were collected. The prevalence varied significantly by age group between children greater than 23 months of age to the rest of the age groups (<6 months, 6–11 months, and 12–22 months) (*p* = 0.0046). The most common *R. alphagastroenteritidis* G-genotypes were G3 (38.7%), G2 (25.4%), and G12 (17.2%), with G2 emerging as the predominant strain from June 2023. G3P[8] was the most frequent G–P combination (25%). Its overall prevalence did not change during the pandemic; however, genotype distribution shifted compared to pre-COVID-19 patterns. Sustained surveillance and genomic analyses are essential to monitor evolving strain dynamics and inform vaccine policy.

## 1. Introduction

*Rotavirus alphagastroenteritidis* (*R. alphagastroenteritidis*) is a leading cause of acute gastroenteritis among children under five years globally. *R. alphagastroenteritidis* is associated with more than 258 million diarrhoea cases and an estimated 128,500–215,000 deaths annually [1]. More than 90% of these deaths occur in Sub-Saharan Africa and south-east Asia [1]. Currently, there are four oral *R. alphagastroenteritidis* vaccines pre-qualified by the World Health Organisation (WHO), which have been in use since 2006: RotaTeq (Merck &Co, Inc., Whiteriver, PA, USA), Rotavac (Bharat Biotech International Ltd., Hyderabad, India), Rotarix (GSK, Rixensart, Belgium), and Rotasiil (Serum Institute of India, Pune, India) [2]. Over 120 countries have introduced a *R. alphagastroenteritidis* vaccine as part of their national vaccination schedule, which has led to a reduction in *R. alphagastroenteritidis*-related mortality and diarrhoea hospitalisations in children under five years old in all settings [3]. However, vaccine efficacy and impact are reduced in low- and middle-income countries (LMICs) compared to in high-income countries (HICs) [4].

*R. alphagastroenteritidis* contains eleven segments of double-stranded RNA (dsRNA) surrounded by non-enveloped triple-layered capsid proteins [5]. The genome encodes six structural (VP1, VP2, VP3, VP4, VP6, and VP7) and five to six non-structural proteins (NSP1, NSP2, NSP3, NSP4, NSP5, and NSP6), depending on the strain [5]. VP6 determines the serogroup, with *R. alphagastroenteritidis*, *R. betagastroenteritidis*, and *R. tritogastroenteritidis* being identified in humans, of which *R. alphagastroenteritidis* is the most prevalent [5]. *R. alphagastroenteritidis* is further differentiated into serotypes, determined by the reactivity of antibodies to capsid surface proteins, VP4 (P serotype) and VP7 (G serotype), or as genotypes by sequencing. The classification system that assigns G and P as genotypes is based on nucleotide sequence similarity of the genes encoding VP7 and VP4 respectively [5].

The G1P[8] Rotarix vaccine was introduced into Malawi’s national Expanded Programme on Immunisation (EPI) in October 2012 [6]. Rotarix coverage exceeded 95% by 2015 [6] and has been associated with the reduced severity of gastroenteritis in vaccinated children compared to in unvaccinated children, regardless of *R. alphagastroenteritidis* genotype [7]. Early post-vaccine introduction studies indicated that the vaccine reduced hospital admissions by 43.2%, with a vaccine effectiveness of 70.7% against the G1 genotype [6]. Despite this, *R. alphagastroenteritidis* remains the most detected pathogen in hospitalised children with diarrhoea-associated disease in Malawi [8].

Malawi experienced four waves of coronavirus disease 2019 (COVID-19), with the first case registered on 2 April 2020 [9]. To manage COVID-19 effectively, Malawi declared a state of national disaster on 20 March 2020 [10]. During this period, non-pharmacological interventions, such as physical distancing, travel restrictions, and increased emphasis on hand hygiene, were introduced, which are potentially effective in reducing infectious diseases, including acute gastroenteritis [11]. Understanding changes in *R. alphagastroenteritidis* genotypes and their clinical presentation during this time is critical, as vaccine efficacy can be influenced by the heterogeneity of the circulating strains [6,12]. This study aimed to evaluate the impact of COVID-19 on *R. alphagastroenteritidis*-associated hospital presentations and genotype distribution with a particular focus on the resurgence of G2 strains in Malawi.

## 2. Materials and Methods

### 2.1. Rotavirus Alphagastroenteritidis Detection and Genotyping

Stool samples were collected from children under five years old who presented with acute gastroenteritis at Queen Elizabeth Central Hospital (QECH) from October 2019 to October 2024. Eligible children had experienced at least three stools that were looser than usual within a single 24-h period for less than seven days. Upon receipt of the stool samples, a 20% stool suspension was prepared in phosphate-buffered saline to screen for the presence of *R. alphagastroenteritidis* using a commercial enzyme immunoassay, EIA (Rotaclone, Meridian-Bioscience, Cincinnati, OH, USA). Another aliquot of raw stool samples was stored at −80 °C and later used to prepare 20% suspension in phosphate-buffered saline for molecular assays. In brief, viral dsRNA from *R. alphagastroenteritidis*-positive stool suspensions was extracted using the Viral RNA Mini-Kit (Qiagen, Hilden, Germany). Superscript III MMLV-RT (Invitrogen, Paisley, UK) and random primers (Invitrogen, Paisley, UK) were used to reverse transcribe the dsRNA to complementary deoxyribonucleic acid (cDNA). The nested RT-PCR was used to determine the G genotypes (G1, G2, G3, G4, G8, G9, G10, G11, and G12) and P genotypes (P[4], P[6], P[8], P[9], P[10], and P[11]) for *R. alphagastroenteritidis*-positive samples, as described previously [13].

### 2.2. Data Analysis

Data were stratified into gender and age groups to determine the proportion of *R. alphagastroenteritidis*-positive cases in different categories. Ages were presented in months (from date of birth to date of sample collection) and shown as total positive cases (all age groups 0–59 months) as well as in range of under 6 months, 6–11 months, >11–23 months, and >23 months. To determine whether there was a difference in the proportion of positive cases per age group, a Pearson chi-squared (χ^2^) test was performed with a level of significance set at 0.05. Median ages and ranges of children who had both positive and negative *R. alphagastroenteritidis* results were calculated, as well as the interquartile range. Mood’s median test was performed on the ages of children who tested positive or negative to determine if this factor was significantly different. The median age of children who tested positive or negative was determined. The number of observations greater than the overall median was determined and a contingency table was generated with the data. A chi-squared test was completed on the contingency table, and the calculated value was compared with the chi-squared critical value.

When both G and P genotypes were detected in a sample, it was classified as a distinct genetic variation and recorded as the presence of the relevant genotype. The number of times each variation appeared was calculated and stratified per age group and presented as a percentage of the total samples in which the genotype was obtained; X/n where X = number of times the genotype was detected and n = total number of samples where a genotype was obtained across all age groups and stratified for each age range, as stated previously. In instances where more than one genotype was detected in a stool sample, each distinct genotype was recorded separately, regardless of whether that stool sample contained heterogenous genotypes. For genotypes with very small counts, no inferential test was performed as results were presented descriptively. Data was organised into positive cases related to total samples taken for each month, October 2019–October 2024. The percentage of cases that were positive (out of the total samples analysed) was calculated and shown on a month-by-month basis, from October 2019 to October 2024. For the determination of vaccination status, the total number of children whose vaccination records were available was considered, and the number of children who had received at least one dose was calculated. Consideration was also made for children who were not yet eligible for the vaccine. Analysis was performed using Microsoft Excel version 16.55 (211111400) and R (version 4.4.1).

The Shannon Diversity Index (H′) was used to quantify the diversity of *R. alphagastroenteritidis* genotypes across seasons and years. The index was computed manually in R (version 4.4.1) using a custom function implementing the standard Shannon formula: *H*′ = −∑_i₌1_^s^ p_i_ ln(p_i_), where *p_i_* denotes the proportional frequency of each genotype and *S* represents the total number of genotypes detected within a given season and year. Diversity analyses were performed separately for VP7 (G types), VP4 (P types), and combined G + P genotypes. Only years with complete data for all three climatic seasons, Cold dry (May–August), Hot dry (September–December), and Hot wet (January–April), were included. Seasonal differences in genotype diversity were assessed using the Kruskal–Wallis test, and pairwise seasonal comparisons were conducted using the Wilcoxon rank sum test.

### 2.3. Ethical Approval

Ethical approval was obtained from the National Health Sciences Research Committee, Lilongwe, Malawi (# 867), and the University of Liverpool Research Ethics Committee, UK (# 000490).

## 3. Results

### 3.1. Demographic Characteristics of Study Participants

A total of 1135 children were enrolled. The median age of the participants was 12 months with an interquartile range (IQR) of 8–18 months. Of the enrolled participants, 9.7% (*n* = 110/1135) were children under six months old, 37.5% (*n* = 426/1135) were between six and eleven months, 36.3% (*n* = 412/1135) were between twelve and twenty-three months, and 16.2% (*n* = 184/1135) were children over twenty-three months (Table 1). Three samples (0.26%) had no age data and, hence, were not categorised and were excluded from subsequent analyses that required age-grouping (Table 1). There were no significant differences in the sex distribution of the study population, although there were more males (661, 58.3%) than females (474, 41.7%) (Table 1). Among *R. alphagastroenteritidis*-vaccine-eligible children, at least 90.7% (*n* = 1029/1135) had received two doses of the Rotarix vaccine, while 2% had received one dose of Rotarix *R. alphagastroenteritidis* vaccine, indicating a high vaccine coverage in the study population (Supplementary Figure S1).

#### Detection of *R. alphagastroenteritidis* in Different Age Groups During and After the COVID-19 Period

Out of 1135 stool samples collected, 29.0% (*n* = 330/1135) tested positive for *R. alphagastroenteritidis* (Table 1). The median age for *R. alphagastroenteritidis*-positive cases was 11 months with IQR 8–15.25 months, while the median age for *R. alphagastroenteritidis*-negative cases was 12 months, with an IQR of 9–20 months. There were no significant differences in the median age between the two groups (*p* > 0.05). The highest *R. alphagastroenteritidis* detection rates were observed in children under 6 months (32.7%, 95% CI: 25.8–40.8) and those aged 6–11 months (32.2%, 95% CI: 26.5–8.5) (Table 1). Conversely, children older than 23 months had the lowest *R. alphagastroenteritidis* detection rate (18.1%, 95% CI: 13.1–24.3) (Table 1). When we compared *R. alphagastroenteritidis* positivity between the age groups, children greater than 23 months of age had significantly lower detection rate compared to younger age groups (<6 months, 6–11 months, and >11–23 months) (*p* < 0.05).

### 3.2. Seasonality of R. alphagastroenteritidis Infection

**Figure 1:**
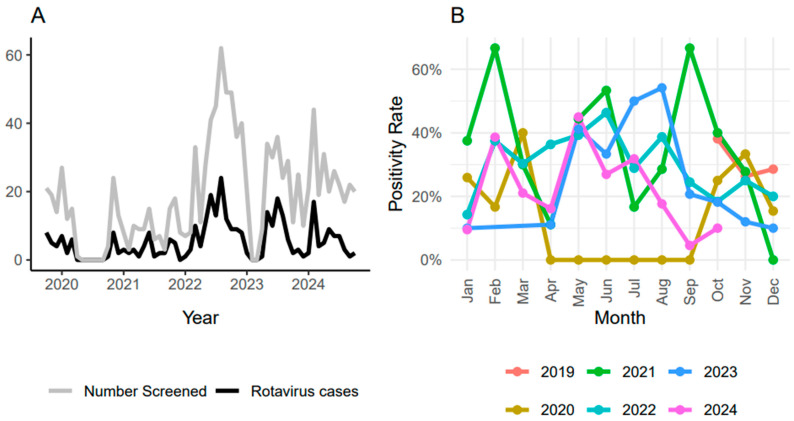
(**A**) Number of positive cases (black line) and number of cases screened (grey line) each year throughout the study. (**B**) Seasonal variation in positivity rate across the years, with some months having more cases than other months. Positivity rate was calculated as the proportion of *R. alphagastroenteritidis*-positive samples among the total screened each year.

Overall, a similar trend was observed between the number of cases screened and those detected, with the number of cases peaking when screening was high (Figure 1A). The number of *R. alphagastroenteritidis* cases detected was highest in the cooler dry months (May to September), except in 2021, where the cases also peaked during winter months, particularly from January to March (Figure 1B). Sample collection was suspended between April and September 2020, following COVID-19 restrictions. During 2020, the percentage of *R. alphagastroenteritidis*-positive cases peaked at 34.8% in November and dropped to 15% in December. During 2021, the percentage of *R. alphagastroenteritidis*-positive cases varied from 15% in December 2020, to 66.7% in February and September 2021. During 2022, *R. alphagastroenteritidis*-positive cases varied from 16.7% in January to 46.3% in June. The highest peak in 2023 occurred in August, when 54% of the *R. alphagastroenteritidis*-positive cases were detected. Similarly, the percentage of positive cases varied from 38% in February to 45% in May 2024. Overall, *R. alphagastroenteritidis* cases exhibited distinct seasonal peaks across the study period.

### 3.3. Temporal Shifts in R. alphagastroenteritidis Genotypes During and After COVID-19

**Figure 2:**
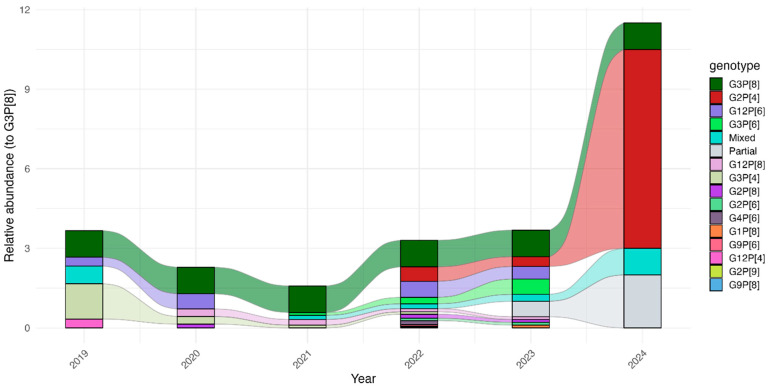
Temporal relative abundance of detected *R. alphagastroenteritidis* strains in relation to G3P[8] strains. Each coloured band represents the abundance of that genotype in relation to the G2P[8] genotype that was consistently detected from 2019 to 2024. A marked shift was observed after 2023, with G2P[4] progressively replacing G3P[8] as the predominant genotype.

Over the course of the surveillance period, we identified 14 distinct *R. alphagastroenteritidis* strains (Table 2). The most common genotypes were G3P[8] (*n* = 83, 25.2%), G2P[4] *(n* = 59, 17.9%), G12P[6] (*n* = 34, 10.3%), and G3P[6] (*n* = 21, 6.4%). G3P[8] was mostly detected in children aged 6 to 11 months. The period from 2019 to 2021 showed a consistent pattern of genotype diversity, with multiple genotypes circulating. The diversity of strains fluctuated, particularly during the pandemic period. Throughout the study, G3P[8] was the most frequently detected strain. However, G2P[4] became predominant, especially from April 2022, and continued to dominate until the end of the study period (Figure 2). No seasonal differences were observed in terms of the genetic diversity of *R. alphagastroenteritidis* genotypes (Supplementary Figure S2, Supplementary Table S1).

## 4. Discussion

*R. alphagastroenteritidis* was consistently detected in Blantyre, Malawi, throughout the study period, except in December 2021, when no positive cases were recorded. Over the five years of surveillance, important insights into *R. alphagastroenteritidis* prevalence and genotype diversity were obtained. The overall detection rate of 29.1% (329/1135) was comparable to previous findings at QECH, Blantyre, with 32.8% (1226/3740) between 1997 and 2007 [14], and 29.6% (934/3155) in the post-Rotarix-vaccine period, 2012 to 2019.

During the COVID-19 pandemic, the Malawi government implemented broad containment measures, including the closure of schools and restrictions on large gatherings [15]. When investigating the risk perception regarding COVID-19 in major cities such as Blantyre, perceptions of COVID-19 risk were shaped by religious beliefs and political opinions linked to concurrent social and political unrest, influencing people’s willingness to adopt non-pharmacological interventions [16]. These socio-cultural factors may have led to a reduced adherence to non-pharmacological measures when compared to countries like Bangladesh, where a high level of compliance with the strict measures was generally observed [17]. The limited adherence to non-pharmacological measures together with continued community interactions likely minimised disruptions to the transmission pathways of *R. alphagastroenteritidis*. Hence, a reduction in *R. alphagastroenteritidis* transmission may not have occurred in Malawi to the extent that it did in other countries. Despite these social challenges, the *R. alphagastroenteritidis* vaccine coverage remained relatively high compared to countries like Tanzania and Mozambique, where vaccine coverage in some regions was significantly affected [18,19].

Seasonal patterns mirrored previously reported trends, with *R. alphagastroenteritidis* detected year-round and peaking during the cooler, drier months [14]. Peaks in positive cases coincided with peaks higher numbers of children screened, likely reflecting periods of increased diarrheal illnesses and greater hospital attendance. Although seasonal peaks were evident, they did not correspond to changes in the genetic diversity of *R. alphagastroenteritidis* strains.

A significant association was observed between age and *R. alphagastroenteritidis* detection, with children under 11 months experiencing the higher burden of *R. alphagastroenteritidis* diarrhoea (32.2%), which is consistent with waning maternal antibodies. This aligns with previous reports showing that severe *R. alphagastroenteritidis* diarrhoea disproportionately affects infants under one year of age [7]. However, 17.4–30.2% of positive samples in this surveillance were from children over 11 months, consistent with earlier studies conducted in Malawi, where 13.6–23.2% of cases occurred in children over 12 months [14,20]. Historically, this age-related pattern has been attributed to immune system immaturity, limited prior exposure, and differences in gut microbiota among younger infants [7].

During the surveillance period, G3P[8] was the most frequently detected genotype, occurring across all age groups. This contrasts with earlier findings in Malawi, where G3P[8] was absent in children under 6 months or over 23 months [13]. G3 strains paired with P[4], P[6], and P[8] were common from 1997 to 1999, re-emerging in 2017, and predominating until 2019 [13]. Whole genome analysis indicated that these re-emerged strains were genetically distinct from the 1990s variants, suggesting importation from abroad. Cross-border migration, which also contributed to the spread of COVID-19 in Malawi [21], may have played a role. Notably, G3 strains disappeared during the pandemic, particularly from June 2024. Similarly, G12—especially G12P[6], a genotype that has been increasingly predominant in Africa since Rotarix introduction [22]—was rarely detected after 2023.

An unexpected finding was the frequent detection of G2 strains, present in 23.9% of *R. alphagastroenteritidis*-positive samples from April 2022. G2P[4] had been a transiently predominant strain in Malawi before Rotarix introduction but declined steadily thereafter and was undetected after 2018 [23]. Similar post-vaccine resurgences of G2P[4] have been reported in South Africa and other regions, showing spatial and temporal variation in strain predominance under different vaccine programmes [24,25]. The reappearance of G2 strains after the COVID-19 era may reflect novel introductions via importation or population movements. In addition, although the G1P[8]-based vaccine provides broad protection, it offers lower protection against heterotypic rotavirus strains, such as G2s, rendering them a replication advantage over homotypic strains like G1P[8] rotavirus, which could explain the surge in G2 rotavirus strains [26]. Sequence analysis of these strains in the present study would help clarify whether their circulation is due to local transmission or international importation. This is a key question, as the past re-emergence of G3 strains has been linked to human mobility, which has driven *R. alphagastroenteritidis* dynamics globally [13]. Ongoing surveillance in Malawi and elsewhere remains critical, given that vaccine efficacy can vary across genotypes, as shown both locally [27] and globally [4].

This study has some limitations. The COVID-19 lockdown disrupted research activities, leading to a gap in sampling between April and September 2020. As a result, the earliest phase of the pandemic is not represented. Nonetheless, all samples collected during the second, third, and fourth COVID-19 waves were analysed, providing a robust characterisation of the pandemic period. Although laboratory-related factors such as RNA degradation may have contributed to the “Untypable” results, other explanations are also possible. The “Untypable” samples could have arisen from a low viral load, primer mismatches, or they were novel genotypes of which may have affected the observed genotype distribution.

## 5. Conclusions

Overall, the prevalence of *R. alphagastroenteritidis* remained stable during and after the COVID-19 pandemic. However, our study revealed a marked shift in genotype distribution, with G2P[4] becoming the predominant genotype, replacing G3P[8] as the dominant genotype during the study period. The G2 resurgence, which was followed by an apparent disappearance of G3 strains, was evident in the post-pandemic period, implying altered genotype dynamics in the aftermath of the pandemic.

These shifts highlight the importance of continued surveillance to assess the longer-term epidemiological impact.

## Figures and Tables

**Table 1:** Summary of age distribution and *R. alphagastroenteritidis* positivity in different age groups.

Age Group	Sample Size (%)	Positive (%)	% Positive Within Age Group	% of All *R. alphagastroenteritidis*-Positive Samples
<6 months	110 (9.7)	36	32.7%	10.9%
6–11 months	426 (37.5%)	137	32.2%	41.5%
>11–23 months	412 (36.3%)	124	30.1%	37.6%
>23 months	184 (16.2%)	32	17.4%	9.7%
NA	3 (0.3%)	1	33.3%	0.3%

**Table 2:** Distribution of *R. alphagastroenteritidis* G and P genotypes detected among positive samples between October 2019 and October 2024 at Queen Elizabeth Central Hospital (QECH) in Blantyre, Malawi.

	P[4]	[6]	P[8]	P[9]	P-Mix	Untypable	Total
G1	-	-	2 (0.6%)	-	-	-	2 (0.6%)
G2	59 (17.9%)	8 (2.4%)	8 (2.4%)	1 (0.3%)	3 (0.9%)	7 (2.1%)	86 (26.1%)
G3	11 (3.3%)	21 (6.4%)	83 (25.2%)	-	2 (0.6%)	14 (4.2%)	131 (39.7%)
G4	-	5 (1.5%)	-	-	-	-	5 (1.5%)
G9	-	2 (0.6%)	1 (0.3%)	-	-	-	3 (0.9%)
G10	-	-	-	-	1 (0.3%)	-	1 (0.3%)
G12	1 (0.3%)	34 (10.3%)	12 (3.6%)	-	2 (0.6%)	9 (2.7%)	58 (17.6%)
G-mix	3 (0.9%)	3 (0.9%)	4 (1.2%)	-	-	0 (0%)	10 (3.0%)
Untypable	1 (0.3%)	5 (1.5%)	6 (1.8%)	1 (0.3%)	-	21 (6.4%)	34 (10.3%)
Total	75 (22.7%)	78 (23.6%)	116 (35.2%)	2 (0.6%)	8 (2.4%)	51 (15.5%)	330 (100%)

## Data Availability

Data generated or analysed during the study period is available upon request to the principial investigator due to ethical reasons.

## Supplementary Materials

The following supporting information can be downloaded at: https://www.mdpi.com/article/10.3390/pathogens14111169/s1, Figure S1: Rotarix *R. alphagastroenteritidis* vaccine coverage among the 1135 recruited study participants; Figure S2: Seasonal variation in Shannon diversity (H′) of *R. alphagastroenteritidis* genotypes in Malawi from October 2019 to October 2024; Table S1: Seasonal Shannon diversity (H′) of *R. alphagastroenteritidis* genotypes in Malawi, 2019–2024.
