# Supplementary material for: Distribution of Rotavirus alphagastroenteritidis Strains in Blantyre, Malawi, During and After the COVID-19 Pandemic
Source: Pathogens. 2025 Nov 16;14(11):1169. doi: 10.3390/pathogens14111169 (PMC12655356; doi:10.3390/pathogens14111169)
Supplement: Supplementary file 1 [file pathogens-14-01169-s001.zip › Supplemantary Table S1.pdf]

Table S1: Seasonal Shannon diversity (H′) of R. *alphagastroenteritidis* genotypes in Malawi, 2019–2024

Type	Season	Mean_H	sd_H	n_years	p
G (VP7)	Cold dry	1.065	0.427	4	0.472
G (VP7)	Hot dry	1.091	0.751	4	0.472
G (VP7)	Hot wet	0.753	0.334	4	0.472
G + P	Cold dry	1.785	0.471	4	0.263
G + P	Hot dry	1.792	0.789	4	0.263
G + P	Hot wet	1.089	0.666	4	0.263
P (VP4)	Cold dry	1.182	0.266	4	0.221
P (VP4)	Hot dry	1.025	0.291	4	0.221
P (VP4)	Hot wet	0.698	0.332	4	0.221
