# Supplementary figures and images for: Distribution of Rotavirus alphagastroenteritidis Strains in Blantyre, Malawi, During and After the COVID-19 Pandemic
Source: Pathogens. 2025 Nov 16;14(11):1169. doi: 10.3390/pathogens14111169 (PMC12655356; doi:10.3390/pathogens14111169)

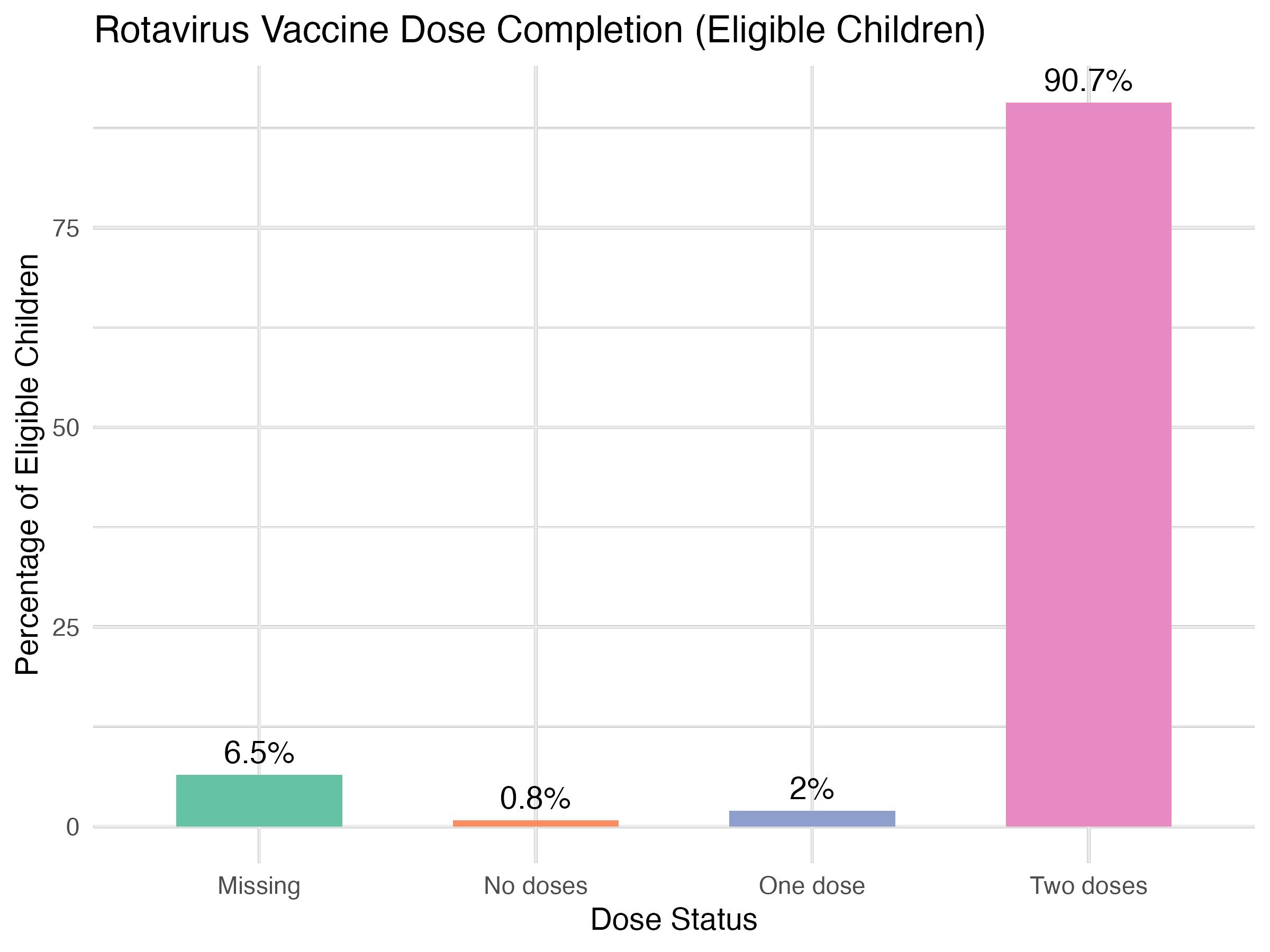

Supplement: Supplementary file 1 [file pathogens-14-01169-s001.zip › Figure S1.tiff]

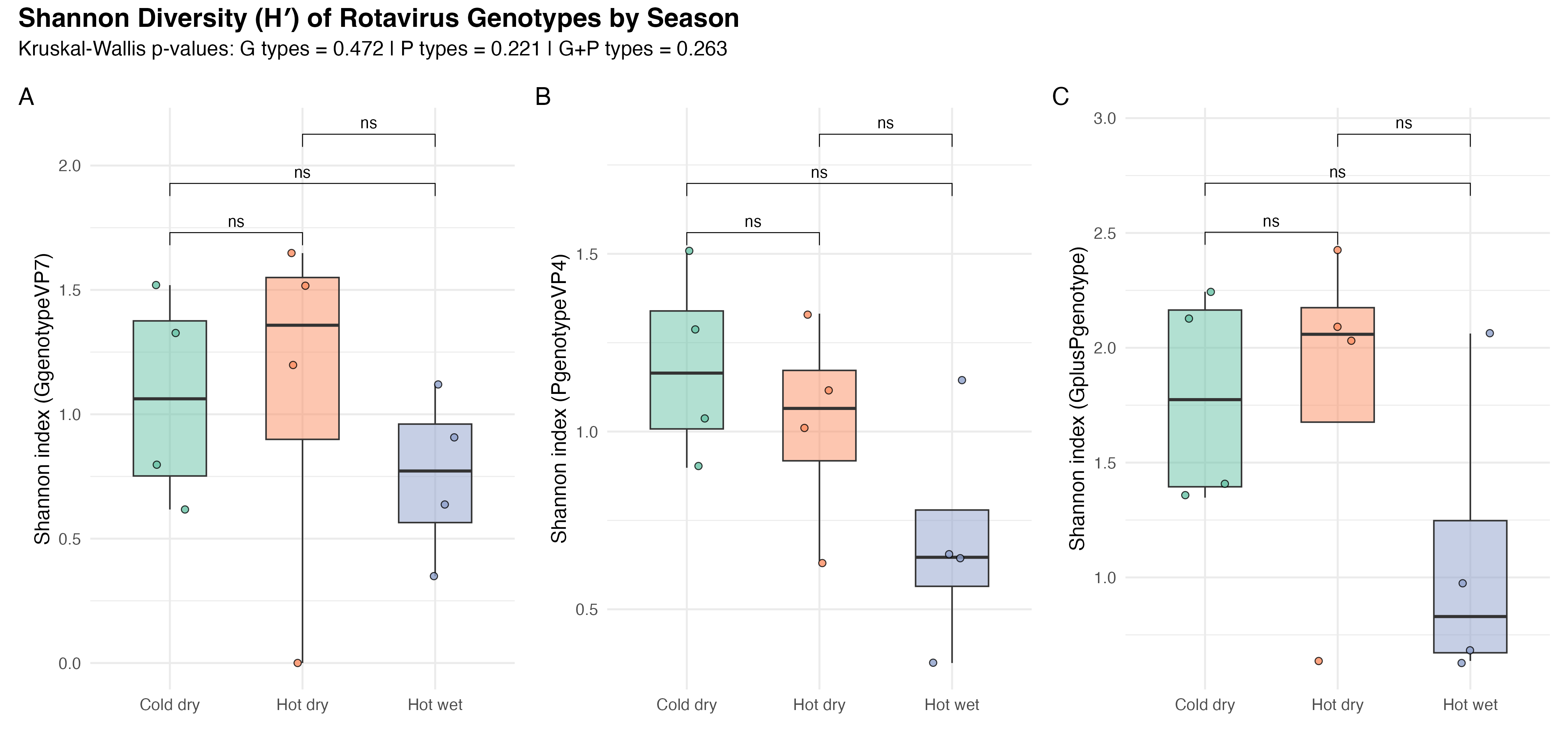

Supplement: Supplementary file 1 [file pathogens-14-01169-s001.zip › Figure S2.tiff]
